# Nutrient Intake and Dietary Adequacy Among Rural Tanzanian Infants Enrolled in the Mycotoxin Mitigation Trial

**DOI:** 10.3390/nu17010131

**Published:** 2024-12-31

**Authors:** Rosemary A. Kayanda, Neema Kassim, Francis M. Ngure, Rebecca J. Stoltzfus, Erica Phillips

**Affiliations:** 1 Department of Food Sciences and Biotechnology, School of Life Sciences and Bioengineering, The Nelson Mandela African Institution of Science and Technology (NM-AIST), Arusha P.O. Box 447, Tanzania; 2 Division of Nutritional Sciences, Cornell University, 244 Garden Ave, Ithaca, NY 14853, USA; 3 Office of the President, Goshen College, 1700 S Main St, Goshen, IN 46526, USA; 4 Department of Nutritional Sciences, University of Wisconsin-Madison, 1415 Linden Dr, Madison, WI 53706, USA

**Keywords:** nutrient intake, complementary feeding, 24 h dietary recalls

## Abstract

Background: The Mycotoxin Mitigation Trial (MMT) was a community-based cluster-randomized trial designed to assess the effect of dietary aflatoxin (AF) on linear growth. Similar dietary intake between arms was an important component of the trial’s program theory and essential for the trial’s internal validity and interpretation. Objective: This analysis assessed and compared dietary intake by arm within a sub-sample of infants enrolled in the MMT. Methods: Twenty paired clusters (10 per trial arm) out of the 52 MMT clusters were included in this sub-sample. Up to 15 maternal/infant dyads per cluster were randomly selected for a one-time, structured, multi-pass 24 h dietary recall. Data were collected at the midpoint of the trial, when infants were 12 months of age, over 8 calendar months. We evaluated and compared infant nutrient intake and adequacy of energy, protein, lipid, iron, zinc, calcium, and vitamin A between study arms. Nutrient intake by arm was estimated using mixed-level regression models. Results: A total of 282 mothers participated (n = 140 intervention arm and 142 standard of care (SoC) arm). The mean daily intakes of energy and lipid fed to infants were 505 kcal/day (SD = 225.9) and 13 g/day (SD = 6.9), respectively, in the intervention and SoC arms, with no difference between arms. Intervention infants consumed slightly more protein than SoC infants (13.7 v. 12.3 g/day, *p* = 0.02). Consumption of iron, zinc, calcium, and vitamin A were low and did not differ between arms. Conclusions: At the midpoint of the MMT, energy, lipid, and micronutrient intake did not differ between arms. Protein consumption was slightly greater in the intervention arm. Guided by the trial’s program theory, this analysis advances the interpretation of the MMT trial findings.

## 1. Background

Stunting is an underlying cause of 3.1 million, or 45% of global child deaths per year [1]. About 148.1 million children under the age of 5 suffered from stunting in 2022, 43% of whom lived in Africa [2]. These numbers may have substantially increased due to constraints in accessing nutritious diets and essential nutrition services due to the COVID-19 pandemic [2,3]. Stunting has adverse consequences on multiple health and developmental outcomes, especially in the first 1000 days from birth to two years [4,5,6].

Aflatoxins (AF) are a family of toxins produced by *Aspergillus flavus* and *A. parasiticus* on food crops such as maize (corn), groundnuts, and tree nuts [7]. Numerous observational studies have documented an association between AF consumption and stunting in infants and young children in low-income countries [8,9,10]. More recent studies have shown mixed results and the only experimental evidence about this relationship is inconclusive [11,12,13,14].

To establish experimental evidence of the possible link between AF exposure and child growth, our research team designed and implemented the Mycotoxin Mitigation Trial (MMT), a cluster randomized trial conducted in the Kongwa District of Tanzania [15,16]. We hypothesized that frequent AF consumption during the complementary feeding period contributes to reduced length for age Z-scores (LAZ). Our primary research question for the MMT was whether the provision of low-AF flours would increase LAZ at 18 months if feeding behaviors were otherwise not altered. Consumption of similar dietary intake between arms was critical to the internal validity of the trial so that if a difference in growth was detected between arms, it could be attributed to AF consumption and not dietary intake. Prior to the conduct of the trial, we used program theory to design a “program impact pathway” [17,18,19]. Dietary intake was a component of the trial’s program theory but was not a primary or secondary outcome of the trial.

The objective of this analysis was to compare the nutrient intake and adequacy of macronutrients and select micronutrients (iron, zinc, calcium, and vitamin A) between the intervention and standard of care (SoC) arms of the MMT and to explain factors that affect dietary intake.

In this analysis, we aimed to answer these questions within a sub-sample of MMT participants:

(1) Did dietary intake differ between study arms? If so, how?
(2) In the context of a nutrition Infant and Young Child Feeding (IYCF) education intervention delivered by MMT-trained community health workers, did adequacy of nutrient intake differ by arm?
(3) Did seasonality significantly affect dietary intake of infants?

## 2. Materials and Methods

### 2.1. Study Background and Population

#### Mycotoxin Mitigation Trial Intervention

Infant/mother dyads were recruited into the MMT when infants were between 6 weeks and 3 months of age. We used Expanded Program on Immunization (EPI) attendance records from each health facility in monthly intervals over 11 months (April 2019–February 2020) to identify eligible infants. Information about inclusion and exclusion criteria can be found in our trial protocol [15,20]. Following enrollment, participants in both arms were invited to four IYCF education sessions led by MMT-trained community health workers. This nutritional education was designed to be age-appropriate for babies in the first year of life and was delivered equally in the two arms.

When the infant turned 6 months of age, the age when complementary feeding is recommended to begin, the randomized intervention was introduced to the mother. Participants in the intervention arm were provided with pre-blended porridge flour, locally called *lishe*, made of low-AF maize and groundnut flour, with a ratio of four parts maize to one part groundnut, the median ratio of porridge blends fed to infants in our formative trials. Separate low-AF groundnut flour was also provided in the intervention arm monthly. These flours were processed by Halisi Products Limited, a local food producer in Arusha, Tanzania [21]. Participants in the SoC arm were provided with skin lotion monthly, a locally desirable product of similar economic value. While 4:1 porridge was promoted to all MMT participants, porridge flours were acquired by the household in the SoC arm.

### 2.2. Sub-Sample Cluster and Participant Selection

At the midpoint of the trial, as infants turned 12 months of age, we randomly selected 284 MMT-enrolled dyads within 20 of the 52 MMT health facility clusters (10 per arm) to participate in this sub-study. These clusters were selected based on the researchers’ ability to access both the catchment point (health facility) and households within clusters in this rural district. We selected this time point because most children at this age consume a variety of family foods in addition to porridge, contributing to their growth outcomes. The size of this sample was intended to balance the time and resource intensive approach of 24 h dietary recalls with a breath of participants across multiple clusters and was conducted in approximately 10% of MMT dyads. Due to the staggered monthly enrollment of the MMT, infant age was evenly spread throughout the year.

To be considered for participation in this dietary study, infants needed to have participated in the 6-month MMT visit, be between 11–13 months of age, and mothers needed to provide informed consent for additional data collection in addition to the informed consent for the larger MMT trial. Mother/infant dyads were excluded from this study if they dropped out of the MMT by the time they were visited or did not provide informed consent for additional data collection.

### 2.3. Data Collection

One quantitative 24-h dietary recall was performed with each mother. Dietary and nutrient intake can vary substantially by season in this region [22]. To account for this seasonal variation, we conducted data collection in three rounds: Round 1 (July–September 2020), Round 2 (October–November 2020), and Round 3 (December 2020–February 2021). The COVID-19 pandemic caused a 3-month delay in the start of the work and resulted in data collection taking place over 8 calendar months.

For the recall, we used a validated, multi-pass dietary recall method to assist the mother to remember all of the food and drinks the child consumed in the past 24 h [23]. The questionnaire was translated into Kiswahili and pre-tested and refined before it was administered to mothers in the household. The five passes included (1) recalling the foods and drinks consumed; (2) describing the foods consumed, including all single foods and ingredients used in recipes; (3) determining the quantity consumed by the infant; (4) converting portion size to weight equivalents; and (5) reviewing the recall with the mother. For pass 4, the actual food, water, or playdough, was used to estimate weight or volume, depending on the availability of the food and according to the study protocol for each food or beverage. The mother’s own measures or local tools carried by the data collectors, such as spoons, tablespoons, bowls, and cups, were used by mothers to weigh foods or ingredients. The volume of liquid foods, including porridge, juice, soup, and sauce, was measured with a cylinder in milliliters.

### 2.4. Statistical Analysis

Recall data were coded by the researchers using the Harvest Plus Ugandan food composition and recipe tables [24]. Foods unique to Kongwa, primarily sauces to accompany *ugali* (stiff porridge) or rice, were added to the food composition table using Tanzanian or USDA sources. After coding, data were entered into CS dietary software (Harvest Plus and SerPro SA, version 2.0) to generate nutrient intake by infant. These data were then exported to STATA 16 for statistical analysis (College Station, TX, USA). These micronutrients were selected due to their effects on child growth or development [25,26,27].

Nutrients with skewed outcomes were logged for analysis (protein, lipid, calcium, iron, zinc, and vitamin A). The first question of comparing nutrient intake by arm was calculated using mixed models controlling for age, gender, and round of data collection (correlated with season) and accounting for health facility clusters as a random effect (xtmixed command). These covariates were stated *a priori.* Nutrient adequacy was determined by comparing intake to the World Health Organization (WHO) nutrient recommendation and presented as the percent of infants who met the recommendation (question 2) [28]. To compare energy, protein, and lipid intake between season, we report the estimated mean intake by round from the mixed models with the Bonferroni correction for multiple comparisons (question 3).

### 2.5. Ethics Approval

Ethical approval for conducting this research was obtained from Cornell University (Protocol #1809008284, 24 October 2018) and the Northern Tanzania Health Research Ethics Committee (KNCHREC) (registration number KNCHREC00041/02/2021, 1 February 2021). All methods were carried out in accordance with relevant guidelines and regulations. Written informed consent was obtained from all mothers to participate in the study; there was one consent procedure for the main trial and a second for those invited into the sub-study.

## 3. Results

### 3.1. Participants

**Figure 1:**
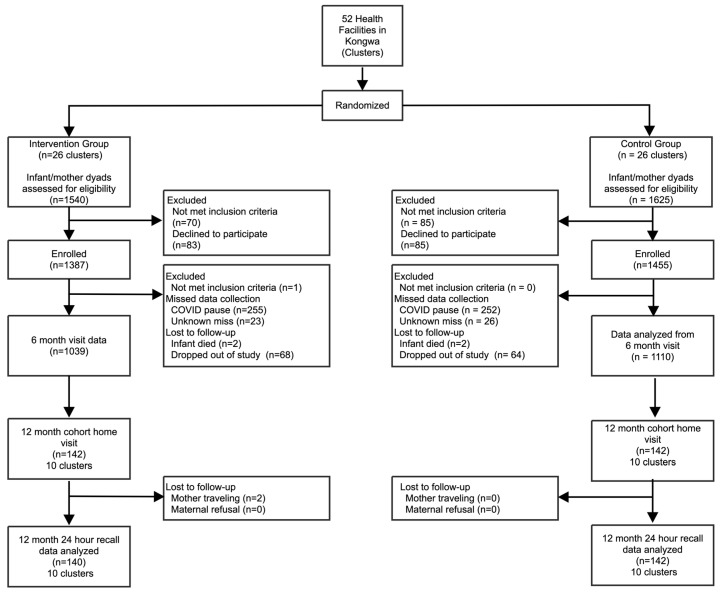
Flow diagram of participants.

We recruited 282 out of 284 maternal/child dyads (n = 140 intervention arm and n = 142 SoC arm) into this sub-study (Figure 1). Two selected participants from the intervention arm were not able to participate due to the travel. All collected data were included in the final analysis.

The mean age of infants in this sub-studywas 11.7 months (range 11–13), with slightly more male infants in both arms (Table 1). Almost 80% of mothers were married, and 95.8% were above 18 years of age, with no differences by arms. Nearly all the participants (99.3% in intervention and 97.2% in SoC arm) were still breastfeeding. Mothers reported that the appetite of the child for the recall period was typical for 66.0% of infants, less than normal for 32.6% of infants, and greater than normal for 1.4% of infants, with no differences by arm.

### 3.2. Nutrient Intake

The mean energy intake was 505 kcal/day (range: 70–1573 kcal/day) and did not differ significantly between arms (Table 2). Mean protein intake was higher in the intervention arm: 13.7 g/day (range: 2.0–45.0 g/day) compared to 12.3 g/day (range: 1.0–46.0 g/day, *p*-value 0.03) in the SoC arm. Zinc and iron intakes were very low and were not statistically significant, although both differences approached significance (Supplementary Table S1).

Because of this differential in protein intake, we compared the feeding episodes of different food groups to understand the drivers of dietary differences between arms. The consumption of roots and tubers, meat, poultry and insects, fish, and seafood together with eggs was similar in both arms. Groundnut consumption was higher in the intervention arm (Supplementary Table S2).

### 3.3. Nutrient Adequacy

The intake for all macro- and micro-nutrients that were evaluated in the study sample was below the recommended nutrient intake (RNI) for breastfed infants. This was particularly true for lipids, iron, and zinc, as less than 10% of infant intakes met the recommended intake (Table 3). For protein and vitamin A, just over 50% of infants overall met the RNI. There were no differences in adequacy between arms for any of these nutrients.

### 3.4. Seasonality

To capture seasonal variability, this study was conducted in three different rounds, each of 2–3 months of duration over 8 months’ time. Round 1 was closest to the harvest months in Kongwa, when a diversity of foods is more available, and Round 3 was furthest from the harvest, when food availability is more limited. This design allowed us to explore nutrient intake differences by season. We found a trend toward higher nutrient consumption during the harvest months (Round 1) compared to later months (Rounds 2 and 3) for all nutrients; however, this only reached statistical significance for lipid (Table 4) and calcium and vitamin A.

## 4. Discussion

This study assessed dietary intake and the adequacy of key nutrients in 282 infants enrolled in the MMT trial to address three research questions. The first question was whether the diets of infants in the two arms were similar, as the trial intervention provided low-AF foods to one arm but not the other, with the same IYCF education delivered to both. The intent of the trial was that diets in both arms would be similar, to create a contrast in AF consumption only. This was found to be true, with one exception. Of the macronutrients and four micronutrients assessed, protein intake differed significantly between arms. Compared to the SoC arm, children in the intervention arm consumed on average 1.6 g more protein daily, which appears to be related to more frequent feeding of groundnuts. Although groundnut consumption was promoted equally between arms in the IYCF education, it is possible the intervention arm consumed more groundnut because this flour was provided by the project.

The second question was whether the nutrient intakes were adequate in this population and to describe any nutrient inadequacies. Slightly more than half of the infants in both arms met the RNI for protein (no difference between arms), and only 20% met the RNI for energy. These results confirmed that the diet was poor in both arms. Inadequate energy and protein can lead to poor growth and brain development and can damage intestinal mucosa and the immune system [30,31].

Among the four micronutrients analyzed, all were generally inadequate with no differences between trial arms. Iron and zinc intake were extremely low in both arms compared to the RNI. Both play essential roles in the healthy growth and development of children and can be difficult to consume in sufficient qualities in LMICs [32,33]. Zinc deficiency in early life increases the risk of morbidity and contributes to poor growth [33]. Iron deficiency can lead to reduced intellectual and motor development [34]. Low consumption of foods rich in bioavailable iron and zinc such as meat, particularly red meat, and high consumption of foods rich in inhibitors of iron and zinc absorption, such as phytate, certain dietary fibers, and calcium, cause iron and zinc deficiencies. These results are similar to findings from central Zimbabwe, which reported low intake of iron (0% met RNI) and zinc (16% met RNI) prior to an intervention with lipid-based nutrient supplement [35]. Improvements in iron and zinc intake are likely to require the promotion of complementary foods, including meat or added supplements [36]. The results from our study confirm the limited intake of meat or beef, as only 5 of 282 (2%) participants reported their consumption.

As part of the IYCF education, the MMT encouraged to continue breastfeeding infants following the introduction of complementary foods through two years of age. Our results show that breastfeeding continued to be high at 12 months, namely 99.3% in the intervention and 97.2% in the SoC, and that intervention-arm mothers maintained typical breastfeeding behavior even when provided with MMT flours.

The third research question pertained to seasonal differences. Seasonal food availability is an important factor that affects nutrient intake and sources [22,37]. Many foods, including groundnuts, fruits, and vegetables, are available during the wet season, but may be unavailable or too expensive to procure during the dry season. Most plant sources of food in Kongwa District are homegrown and are not available throughout the whole year [38]. We observed lower protein, energy, and lipid intakes in December 2020 to February 2021, which only reached statistical significance for lipid intake.

### Strengths and Limitations of the Study

Our study was ambitious in the number of diet recalls achieved. We performed single dietary recalls with all participants; a second recall might have improved the validity of the findings and allowed us to account for intra-individual variation [39]. However, because we had a sample size of 282 infants and balance in key variables related to dietary intake between arms, we believe our comparisons between arms are robust at the group level. This analysis was performed in 282 children out of almost 3000 enrolled in the MMT due to practical constraints in time and resources. We compared key maternal and infant variables in this sub-sample to the full MMT sample and did not find differences between them (Supplementary Table S3).

## 5. Conclusions

This study assessed the nutrient intake and adequacy of infant diets in central Tanzania and compared diets between the SoC and intervention arms of the MMT trial. Answering these three research questions, based on the trial’s program theory, allowed us to carefully interpret the study results and avoid a “black box” study, in which we might see an effect on our outcome of interest but not understand its cause. Comparable dietary intake between the two arms was essential for the internal validity of the trial and was found to be similar except for a difference in protein intake. Even with a locally adapted IYCF education intervention, the percent of infants who met the RNI for all nutrients assessed was low, indicating poor diets in this region, with evidence that dietary stress measured by macronutrient intakes was most extreme in December–February in the calendar years studied.

## Figures and Tables

**Table 1:** Characteristics of mother-infant dyads, by arm.

Variable	Description	Intervention Arm	SoC Arm	*p*-Value
		n = 140	n = 142	
Infant age at 24 h recall (months)	Mean (SD, range)	11.7 (0.5, 11–13)	11.7(0.4,11–13)	0.97
Gender of infant				0.71
	Males	78 (55.7%)	76 (53.5%)	
	Females	62 (44.3%)	66 (46.5%)	
Maternal age (years)	Mean (SD, range)	27.1 (7.4, 16–44)	27.1 (7.6, 16–46)	0.96
Marital status	Currently married	112 (80.0%)	110 (77.5%)	0.60
Maternal schooling				0.40
	No schooling	45 (32.1%)	38 (26.8%)	
	Attended primary	16 (11.4%)	10 (7.0%)	
	Completed primary	68 (48.6%)	78 (54.9%)	
	Attended secondary	4 (2.9%)	8 (5.6%)	
	Completed secondary	7 (5.0%)	8 (5.6%)	
Ethnic group				0.70
	Gogo	74 (52.9%)	71 (50.0%)	
	Kaguru	45 (32.1%)	60 (42.3%)	
	Other	21 (15.0%)	11 (7.7%)	
No. of people in the house	Mean (SD, range)	5.7 (2.1, 3–17)	6.0 (2.2, 2–14)	0.18
Primary drinking water source				0.52
	Piped	99 (70.7%)	116 (81.7%)	
	Dug well	24 (17.1%)	15 (10.6%)	
	Spring water	4 (2.9%)	0 (0.0%)	
	Rain/surface water	2 (1.4%)	0 (0.0%)	
	Other	11 (7.9%)	11 (7.8%)	

Note: Except child age, all characteristics are reported from the MMT recruitment survey. *p*-values reported from *t*-tests are reported for continuous variables and chi-square for categorical variables. Percentages are rounded.

**Table 2:** Estimated daily macro- and micronutrient intake by arm.

Nutrient	Intervention Arm Mean (95% Confidence Interval)	SoC Arm Mean (95% Confidence Interval)	Difference Between Groups (% of Total Sample Mean)	*p*-Value *
	n = 140	n =142		
Energy (kcal)/day	519.7 (481.9, 556.3)	492.2 (455.3, 529.1)	26.9 (5.3%)	0.32
Protein (g)/day	12.3 (11.2, 13.4)	10.7 (9.8,11.6)	1.6 (13.9%)	0.03
Lipid (g)/day	12.2 (11.0, 13.4)	11.0 (9.9, 12.1)	1.2 (10.3%)	0.19
Iron (mg)/day	1.9 (1.7, 2.1)	1.7 (1.5,1.9)	0.2 (11.1%)	0.07
Zinc (mg)/day	1.5 (1.4, 1.7)	1.4 (1.3, 1.5)	0.1(6.7%)	0.06
Calcium (mg)/day	72.1 (59.3, 84.9)	74.9 (61.7, 88.1)	2.8 (3.8%)	0.72
Vitamin A (ug)/day	247.8 (190.6, 305.1)	233.1 (179.6, 286.6)	14.3 (5.9%)	0.78

* *p*-values reported from mixed level models adjusting for age, arm, gender, and round with cluster as a random effect (protein, lipid, iron, zinc, calcium, and vitamin A).

**Table 3:** Percent of infants who met Recommended Nutrient Intake (RNI) by arm.

Nutrient	RNI for 7–12 Months of Age	Intervention Arm % Met RNI	SoC Arm % Met RNI	*p*-Value
		n = 140	n = 142	
Energy (kcal)/day	479 kcal/day	22.9%	21.8%	0.84
Protein (g)/day	10.5 g/day	62.1%	52.8%	0.11
Lipid (g)/day	35% of caloric intake/day	7.1%	7.0%	0.97
Iron (mg)/day	9.3 g/day	1.4%	0.0%	0.15
Zinc (mg)/day	4.1 mg/day	1.4%	0.7%	0.55
Calcium (mg)/day	400 mg/day	3.6%	7.0%	0.19
Vitamin A (ug)/day	400 ug/day	38.6%	32.4%	0.28

Notes: RNI for energy is for complementary foods only and assumes average breastmilk intake by age (9–11 months and 12–23 months) [29]. For non-breastfed infants > 12 months old, we used 858 kcal/day for adequacy; protein—WHO/Food Agricultural Organization) (FAO) requirements: we used the average by age and sex at 10.5 for 12 months and 11.9 > 12 [28]. The values shown are averages across age and sex; for iron and zinc, we assumed moderate bioavailability. There were 4 infants 13 months of age; in this analysis, for energy, protein, and lipids nutrients, the appropriate cutoff for 13-month-olds was used.

**Table 4:** Macronutrient intake by rounds of data collection.

Nutrient	Rounds	Predicted Means	Standard Error	Bonferroni Groups
Energy (kcal)/day	Round 1: July–September 2020	537.5	30.1	A
	Round 2: October–November 2020	522.2	18.8	A
	Round 3: December 2020–February 2021	458.6	23.8	A
Protein (g)/day	Round 1: July–September 2020	12.0	0.9	A
	Round 2: October–November 2020	12.0	0.5	A
	Round 3: December 2020–February 2021	10.3	0.6	A
Lipid (g)/day	Round 1: July–September 2020	12.0	0.9	AB
	Round 2: October–November 2020	12.4	0.6	B
	Round 3: December 2020–February 2021	10.1	0.6	A

Note: Predicted means (margins) reported from mixed-level models adjusting for age, arm, gender, and round with cluster as a random effect. Predicted means sharing a letter in the group label are not significantly different at the 5% level.

## Data Availability

Data supporting the results reported in this paper are publicly available: https://drive.google.com/drive/folders/1go_-AmSr5iG--FrvhQhfR53SEKuraoNH?usp=share (accessed on 26 December 2024). Alternatively, the corresponding author can be contacted for requests.

## Supplementary Materials

The following supporting information can be downloaded at: https://www.mdpi.com/article/10.3390/nu17010131/s1, Table S1: Mixed model regression results for key nutrients; Table S2: Frequency and mean number of feeding episodes by food group by study arm; Table S3: Comparison of full trial sample compared to 24-hour dietary recall sub-sample, by arm.

## Abbreviations

AF	Aflatoxin
FAO	Food Agricultural Organization
IYCF	Infant and Young Child Feeding
KNCHREC	Northern Tanzania Health Research Ethics Committee
LAZ	length for age Z-scores
MMT	Mycotoxin Mitigation Trial
RNI	Recommended Nutrient Intake
SD	Standard deviation
SoC	Standard of care
WHO	World Health Organization
