# Supplementary material for: Nutrient Intake and Dietary Adequacy Among Rural Tanzanian Infants Enrolled in the Mycotoxin Mitigation Trial
Source: Nutrients. 2024 Dec 31;17(1):131. doi: 10.3390/nu17010131 (PMC11722735; doi:10.3390/nu17010131)
Supplement: Supplementary file 1 [file nutrients-17-00131-s001.zip › nutrients-3350696-supplementary.pdf]

Kayanda, R. Nutrient intake and dietary adequacy among rural Tanzanian infants enrolled in the Mycotoxin Mitigation Trial

Supplementary Table S1 - Mixed model regression results for key nutrients

Nutrient	Intercept	Arm	Gender	Age	Round of data collection
Energy	575.4 (77.2)	-26.9 (26.8)	-52.4* (26.3)	57.7* (27.6)	-35.1 (19.0)
Protein	2.55* (0.2)	-0.14* (0.06)	-0.10 (0.06)	0.15* (0.07)	-0.07 (0.05)
Lipid	2.55* (0.19)	-0.14* (0.06)	-0.10 (0.06)	0.15* (0.07)	-0.07 (0.05)
Iron	0.44* (0.21)	-0.13 (0.07)	-0.05 (0.07)	0.16* (0.08)	0.01 (0.05)
Zinc	0.42* (0.18)	-0.11 (0.06)	-0.10 (0.06)	0.15* (0.06)	-0.04 (0.04)
Calcium	4.19* (0.40)	0.05 (0.13)	-0.03 (0.13)	0.38* (0.13)	-0.27 *(0.09)
Vitamin A	6.15*(0.48)	-0.04 (0.17)	-0.11 (0.17)	0.04 (0.17)	-0.27* (0.12)

Notes: n=282 for all models (140 intervention arm, 142 SoC arm)
Standard errors reported in parentheses
Protein, lipid, iron, zinc, calcium and vitamin A were log transformed
* Indicates statistically significant at p<0.05

Supplementary Table S2: Frequency and mean number of feeding episodes by food group by study arm

Ingredient	Arm	Percent of infants who consumed ingredient	Mean number of feeding episodes/day	SD	*p*-value
Blended porridge flour (Lishe 4:1)	Intervention	96%	7.8	5.3	0.49
	SoC	75%	7.3	5.3	
Groundnuts	Intervention	67%	8.1	5.7	0.05
	SoC	44%	6.3	5.6	
Dairy	Intervention	12%	12.2	7.3	0.36
	SoC	25%	10.3	6.6	
Egg	Intervention	1%	11.0	2.8	0.66
	SoC	2%	17.7	18.5	
Beef	Intervention	6%	7.3	3.1	0.70
	SoC	6%	8.1	5.1	
Green peas, yellow and red beans	Intervention	33%	10.4	6.7	0.18
	SoC	27%	8.4	7.2	
Fish	Intervention	4%	9.7	7.1	0.35
	SoC	3%	16.8	15.8	

Note: The *p*-value for comparison of mean number of feeding episodes of protein-source ingredients by arm was calculated by two-sample t test with equal variances

Kayanda, R. Nutrient intake and dietary adequacy among rural Tanzanian infants enrolled in the Mycotoxin Mitigation Trial

Supplementary Table S3: Comparison of full trial sample compared to 24-hour dietary recall sub-sample by arm

Variable	Description	Full Trial Intervention Arm	Full trial SOC Arm	Subsample Intervention arm	Subsample SoC arm
		n = 1387	n = 1455	n = 140	n=142
Infant age at 24 hour recall (mos)	Mean (SD, range)	NA	NA	11.7 (0.5, 11-13)	11.7(0.4,11-13)
Gender of infant	Males	664 (47.9)	721 (49.6)	75 (53.6%)	80 (56.3%)
	Females	723 (52.1)	734 (50.4)	65 (46.4%)	62 (43.7%)
Maternal age (years)	Mean (SD, range)	26.4 (7.3, 16-48)	26.5 (7.3, 16-51)	27.1 (7.4, 16-44)	27.1 (7.6, 16-46)
Marital status	Currently Married	1033 (74.5%)	1095 (75.3%)	111 (79.3%)	112 (78.9%)
Maternal Schooling					
	No schooling	441 (31.8%)	501 (34.5%)	45 (32.1%)	38 (26.8%)
	Attended Primary	166 (12.0%)	162 (11.1%)	16 (11.4%)	10 (7.0%)
	Completed Primary	640 (46.1%)	658 (45.3%)	68 (48.6%)	78 (54.9%)
	Attended Secondary	64 (4.6%)	61 (4.2%)	4 (2.9%)	8 (5.6%)
	Completed Secondary	68 (4.9%)	60 (4.1%)	7 (5.0%)	6 (4.2%)
Ethnic Group					
	Gogo	692 (49.9%)	547 (37.6%)	74 (52.9%)	71 (50.0%)
	Kaguru	504 (36.4%)	724 (49.8%)	45 (32.1%)	60 (42.3%)
	Other	190 (13.7%)	182 (12.5%)	21 (15.0%)	11 (7.7%)
No of people in the house	Mean (SD, range)	5.9 (2.2, 2-23)	6.1 (2.4, 2-24)	5.7 (2.1, 3-17)	6.0 (2.2,2-14)
Primary drinking water source					
	Piped	929 (67.0%)	1139 (78.3%)	99 (70.7%)	116 (81.7%)
	Dug well	279 (20.1%)	177 (12.2%)	24 (17.1%)	15 (10.6%)
	Spring water	22 (1.6%)	18 (1.2%)	1 (0.7%)	0 (0.0%)
	Rain/surface water	42 (3.0%)	40 (2.7%)	7 (5.0%)	4 (2.8%)
	Other	115 (8.3%)	81 (5.6%)	9 (6.4%)	7 (4.9%)
